# Does intraoperative reduction result in better outcomes in low-grade lumbar spondylolisthesis after transforaminal lumbar interbody fusion? A systematic review and meta-analysis

**DOI:** 10.3389/fmed.2024.1350064

**Published:** 2024-04-12

**Authors:** Rongqing Qin, Min Zhu, Pin Zhou, Anhong Guan

**Affiliations:** ^1^Department of Spinal Surgery, Gaoyou People's Hospital, Yangzhou, Jiangsu, China; ^2^Department of Orthopedics, The Third Clinical Medical College of Yangzhou University, Yangzhou, Jiangsu, China; ^3^Department of Medical Image, Gaoyou People's Hospital, Yangzhou, Jiangsu, China; ^4^Department of Orthopedics, Gaoyou Hospital of Integrated Traditional Chinese and Western Medicine, Yangzhou, Jiangsu, China

**Keywords:** transforaminal lumbar interbody fusion, reduction, arthrodesis *in situ*, spondylolisthesis, meta-analysis

## Abstract

**Objective:**

This study aimed to compare the clinical efficacy and safety of reduction vs. arthrodesis *in situ* with transforaminal lumbar interbody fusion (TLIF) for low-grade lumbar spondylolisthesis.

**Study design:**

Systematic review and meta-analysis.

**Methods:**

A comprehensive literature search was implemented in PubMed, Embase, and Cochrane Library databases. Randomized or non-randomized controlled trials that were published until July 2023 that compared reduction vs. arthrodesis *in situ* techniques with minimally invasive or open-TLIF for low-grade spondylolisthesis were selected. The quality of the included studies was evaluated by the Newcastle–Ottawa Scale (NOS). Data were extracted according to the predefined outcome measures, including operation time and intraoperative blood loss; short- and long-time follow-up of visual analog scale (VAS) back pain (VAS-BP) and Oswestry Disability Index (ODI); slippage and segmental lordosis; and the complication and fusion rate.

**Results:**

Five studies (*n* = 495 patients) were finally included. All of them were retrospective cohort studies with Evidence Level II. The pooled data revealed that both techniques had similar patient-reported outcomes (VAS, ODI, and good and excellent rate) during short- and long-term follow-up. In addition, no significant differences were observed in the fusion and complication rates. However, although the reduction group did achieve better slippage correction, it was associated with increased operation time and intraoperative blood loss compared with the *in situ* arthrodesis group.

**Conclusions:**

Based on the available evidence, intraoperative reduction does not result in better clinical outcomes in low-grade spondylolisthesis after minimally invasive or open-TLIF, and the *in situ* arthrodesis technique could be an alternative.

## Introduction

Lumbar spondylolisthesis is defined as the forward slippage of the superior vertebrae relative to the inferior vertebrae (1). In addition, according to the degree of slippage assessed by radiology, Meyerding proposed that 1%−25% slippage represents Grade 1, 26%−50% represents Grade 2, 51%−75% represents Grade 3, and 76%−100% slippage represents Grade 4; low-grade spondylolisthesis represents from 1% up to 50% slippage (2). In addition, based on the etiology of spondylolisthesis, Wiltse classified it into five types, namely, congenital, isthmic, degenerative, traumatic, and pathologic (3). For patients with symptoms of degenerative or isthmic spondylolisthesis, such as persistent mechanical pain or low back pain and/or overt neurological deficits, surgical management has been confirmed to have a greater advantage than conservative treatment strategies (4–6). To date, various surgical techniques have been performed to deal with symptoms of spondylolisthesis. Furthermore, the main aim is to stabilize the spinal segment and decompress the neural elements (7–9). In 1982, Harms first described the transforaminal lumbar interbody fusion (TLIF) technique (10). Since then, it has gained popularity as an effective management strategy for lumbar spondylolisthesis (11). TLIF reduces the retraction of nerve roots and the thecal sac and preserves the structural integrity of the posterior column (12, 13). The clinical indications for TLIF surgery are as follows: (1) Grade 1 or 2 degenerative or isthmus lumbar spondylolisthesis without accompanying neurological symptoms or with only unilateral neurological symptoms; (2) discogenic lower back pain with ineffective conservative treatment; (3) intervertebral disc herniation accompanied by lumbar instability (including extreme lateral disc herniation); (4) patients with single-segment lumbar disc herniation who require revision after surgery; and (5) formation of false joints between vertebrae. Moreover, with the development of operative instruments, the TLIF technique has developed into a minimally invasive procedure, which was first described by Foley in 2002 (14). With the potential advantages of smaller incisions, less soft tissue trauma, and faster recovery, the minimally invasive TLIF (MIS-TLIF) technique has become more and more popular (15, 16). In addition, the indications for MIS-TLIF are also constantly expanding, while the contraindications are comparatively shrinking. However, no consensus has been reached on the correlation between reduction and clinical outcomes in patients with low-grade spondylolisthesis. Does intraoperative reduction result in better outcomes after TLIF? This study was performed to estimate the clinical efficacy and safety between reduction and arthrodesis *in situ* with MIS/open-TLIF for low-grade spondylolisthesis.

## Materials and methods

### Search strategy

The systematic review and meta-analysis was conducted in adherence to the Preferred Reporting Items for Systematic Reviews and Meta-Analyses (PRISMA) guidelines (17). A comprehensive literature retrieval was implemented in PubMed, Embase, and Cochrane Library databases. Randomized or non-randomized controlled trials published up to July 2023, which compared reduction vs. arthrodesis *in situ* with MIS/open-TLIF for low-grade spondylolisthesis were achieved. For maximum sensitivity of the search strategy, the following terms were used: (1) transforaminal lumbar interbody fusion OR TLIF OR minimally invasive transforaminal lumbar interbody fusion OR MIS-TLIF; (2) reduction OR without reduction OR *in situ*; and (3) spondylolisthesis; and (4) (1), (2), AND (3). The retrieval was limited to studies published in English. Two reviewers screened the titles and abstracts of all identified studies independently, and full-text copies of relevant studies were obtained. Reference lists of the retrieved studies and previous reviews were manually checked to identify additional potentially relevant research studies. Differences between them were resolved by discussion with a third reviewer.

### Inclusion criteria

The inclusion criteria were as follows:

(1) Study design: randomized or non-randomized cohort trial; (2) patients: low-grade spondylolisthesis with mechanical low back pain and/or radiculopathy; (3) intervention measures: reduction vs. arthrodesis *in situ* with TLIF/MIS-TLIF and pedicular screw fixation; and (4) predefined outcome measures: operation time and intraoperative blood loss; short- and long-time follow-up of VAS back pain (VAS-BP) and Oswestry Disability Index (ODI); slippage and segmental lordosis; and the complication and fusion rate.

### Exclusion criteria

The exclusion criteria were as follows:

(1) patients suffering from spinal trauma, infectious diseases, or tumors; (2) those who opted for/were eligible for other operative procedures such as posterolateral fusion (PLF) and posterior lumbar intervertebral fusion (PLIF); (3) those who have experienced previous lumbar surgery; (4) follow-up time of < 1 year; and (5) repeated studies, animal research studies, non-comparative studies, biomechanical or cadaveric studies, case reports, and reviews.

### Data extraction

We extracted data from each study based on the following terms: (1) first author and year of publication; (2) study design; (3) country; (4) patient demographics; (5) surgical procedure; (6) sample size; (7) type and grade of spondylolisthesis; (10) follow-up time; (11) predefined outcome measures: operation time and intraoperative blood loss; short- and long-term follow-up of VAS-BP and ODI; slippage and segmental lordosis; good and excellent rate; and the complication and fusion rate. When the same participants were included in several publications, we retained only the study with the maximum sample size to avoid duplication of dates. In addition, we defined a follow-up that ranged from 3 to 6 months after surgery as a short-term follow-up and a follow-up of at least 2 years as a long-term follow-up.

### Statistical analysis

All statistical analyses were performed using Review Manager Version 5.3 (Cochrane Collaboration), and heterogeneity among studies was calculated using the chi-square test and quantified via calculating *I*^2^ statistic. For the pooled effects, weighted mean difference (WMD) was calculated for continuous variables according to the consistency of measurement units, while relative risk (RR) was used for dichotomous variables. In the present study, the continuous variables were summarized by WMD and 95% confidence intervals (95% CIs), while the dichotomous variables were represented using RR and 95% CIs. For *I*^2^ < 50%, we preferred the fixed-effects model; otherwise, the random-effects model was used. All *P*-values were two-sided, and a *P*-value < 0.05 was considered statistically significant.

## Results

### Search results

**Figure 1:**
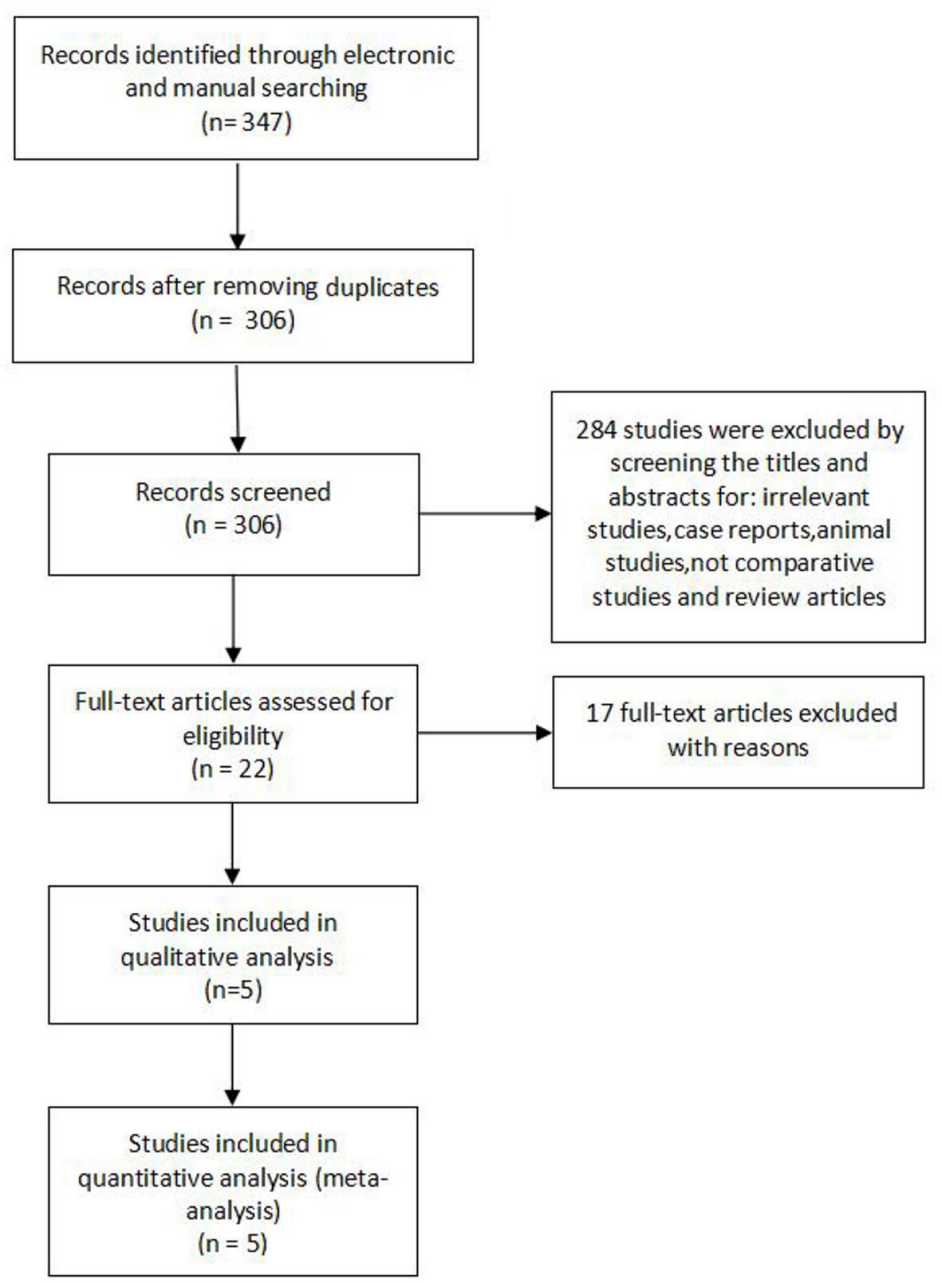
The flowchart showed the process for identifying relative studies.

The flow diagram Figure 1 shows the filtering process for relative studies. A total of 347 studies were initially obtained by electronic and manual searching. According to the inclusion and exclusion criteria that were set previously, five articles (18–22) were finally included for quantitative analysis.

### Baseline characteristics and quality assessment

Five retrospective cohort studies (18–22) included 495 patients (278 in the reduction group and 217 in the arthrodesis *in situ* group) with low-grade lumbar spondylolisthesis. The baseline characteristics of patients in each study are shown in Table 1. The summary of outcome measures for the two interventions is presented in Table 2. Their quality was assessed according to the Newcastle–Ottawa Scale (NOS) (23). In this scale, scores range from 0 (lowest quality) to 9 (highest quality), and the range 7–9 represents good or high quality, while the range 0–3 represents poor or low quality. Two investigators graded the risk of bias of included studies independently. In addition, all studies were rated with a total score of more than 5 (Table 3), signifying relatively moderate to high quality.

**Table 1:** Baseline characteristics of the included studies.

**References**	**Study design**	**Study location**	**Operative technique**	**Sample size and sex (M/F)**	**Mean age (years, range)**	**BMI (kg/m^2^)**	**No. of Spondylolisthesis Grade I and II**	**Follow-up time (months, range)**
Gong et al. (18)	Level II Retrospective	China	TLIF	A: 21 (9/12) B: 13 (6/7)	A: 45.3 ± 31.9 B: 47.1 ± 25.6	NR	A:0/21 B:0/13	A: 30.3 (26–38) B: 28.5 (25–31)
Scheer et al. (19)	Level II Retrospective	USA	MIS-TLIF	A:162 (48/114) B: 120 (36/84)	A: 61.68 ± 10.43 B: 61.88 ± 11.76	NR	A: 97/65 B: 114/6	≥12
Fan et al. (20)	Level II Retrospective	China	MIS-TLIF	A: 41 (10/31) B: 37 (13/24)	A: 60.95 ± 9.06 B: 59.81 ± 9.34	A: 22.70 ± 1.49 B: 22.32 ± 1.86	A: 21/20 B: 23/14	A: 30.78 ± 14.15 B: 28.95 ± 10.75
Fan et al. (21)	Level II Retrospective	China	MIS-TLIF	A: 24 (11/13) B: 21 (10/11)	A: 50.53 ± 12.11 B: 50.05 ± 13.54	A: 22.84 ± 1.62 B: 22.64 ± 1.28	A: 9/15 B: 12/9	A: 34.75 ± 8.06 B: 31.05 ± 6.52
Tay et al. (22)	Level II Retrospective	Singapore	MIS-TLIF	A: 30(11/19) B: 26(5/21)	A: 56.43 ± 11.69 B: 58.28 ± 12.22	A: 25.86 ± 4.36 B: 26.11 ± 3.87	A: 30/0 B: 22/4	≥60

**Table 2:** Summary of outcome measures in the included studies.

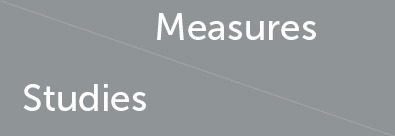	**Gong et al. (18)**	**Scheer et al. (19)**	**Fan et al. (20)**	**Fan et al. (21)**	**Tay et al. (22)**
Operative duration (min)	A: 173.9 ± 23.2 B: 148.4 ± 31.7^§^	A: 228.28 ± 73.63 B: 222.44 ± 60.25^‡^	A: 186.34 ± 46.14 B: 183.24 ± 43.72^‡^	A: 189.58 ± 46.39 B: 178.57 ± 37.19^‡^	A: 173.37 ± 40.09 B: 164.27 ± 51.86^‡^
Intraoperative blood loss (ml)	A: 527.7 ± 205.4 B: 369.2 ± 123.2^§^	A: 280.20 ± 24.03 B: 212.61 ± 17.54^§^	A: 297.56 ± 166.57 B: 281.08 ± 139.12^‡^	A: 279.17 ± 121.51 B: 259.52 ± 102.0^‡^	A: 127.33 ± 61.86 B: 119.23 ± 69.39^‡^
Time to ambulation (day)	NR	NR	NR	NR	A: 1.33 ± 0.72 B: 2.15 ± 4.80^§^
Hospital stay (day)	A: 7.5 ± 3.2 B: 8.1 ± 3.4^‡^	A: 3.79 ± 0.22 B: 3.92 ± 0.21^§^	A: 16.49 ± 4.58 B: 15.43 ± 3.52^‡^	A: 14.71 ± 3.87 B: 15.52 ± 3.25^‡^	A: 3.23 ± 1.18 B: 4.06 ± 4.43^‡^
VAS back pain (range 0–10)	Preop- A: 7.5 ± 1.5 B: 8.0 ± 1.9^‡^ 3-month postoperatively A: 2.4 ± 1.3 B: 2.6 ± 1.8^‡^ Last follow-up (≥2-year) A: 2.2 ± 1.5 B: 1.5 ± 1.4^‡^	NR	Preop- A: 7.17 ± 0.54 B: 7.27 ± 0.69^‡^ 3-month postoperatively A: 3.41 ± 0.63 B: 3.38 ± 0.55^‡^ Last follow-up (≥2-year) A: 2.12 ± 0.68 B: 2.05 ± 0.70^‡^	Preop- A: 7.58 ± 0.58 B: 7.57 ± 0.68^‡^ 3-month postoperatively A: 3.79 ± 0.66 B: 3.90 ± 0.62^‡^ Last follow-up (≥2-year) A: 2.38 ± 0.82 B: 2.29 ± 1.00^‡^	Preop- A: 6.83 ± 2.13 B: 5.08 ± 2.93^‡^ 6-month postoperatively A: 2.88 ± 2.16 B: 2.08 ± 2.15^‡^ Last follow-up (≥2-year) A: 1.2 ± 0.27 B: 0.46 ± 1.09^‡^
ODI (%)	Preop- A: 53.8 ± 19.2 B: 55.0 ± 18.0^‡^ 3-month postoperatively A: 16.2 ± 8.9 B: 15.6 ± 11.1^‡^ Last follow-up (≥2-year) A: 15.2 ± 7.5 B: 13.2 ± 5.8^‡^	NR	Preop- A: 48.59 ± 9.55 B: 50.22 ± 9.91^‡^ 3-month postoperatively A: 27.51 ± 8.33 B: 29.62 ± 9.36^‡^ Last follow-up (≥2-year) A: 17.17 ± 5.73 B: 17.24 ± 4.70^‡^	Preop- A: 51.58 ± 14.12 B: 50.43 ± 11.75^‡^ 3-month postoperatively A: 29.52 ± 11.67 B: 28.48 ± 5.69^‡^ Last follow-up (≥2-year) A: 19.33 ± 7.14 B: 18.10 ± 7.28^‡^	Preop- A: 47.40 ± 12.51 B: 39.16 ± 13.70^‡^ 6-month postoperatively A: 20.94 ± 10.29 B: 16.87 ± 9.05^‡^ Last follow-up (≥2-year) A: 11.28 ± 5.40 B: 8.92 ± 4.75^‡^
Fusion rate (%)	A: 95.2 (20/21) B: 92.3 (12/13)^‡^	A:84.57 (137/162) B:70.83 (85/120)^§^	A: 92.7 (38/41) B: 81.1(30/37)^‡^	A: 91.7 (22/24) B: 85.7 (18/21)^‡^	A: 100 (30/30) B: 100 (26/26)^‡^
Slippage (%)	Preop- A: 39.1 ± 5.9 B: 41.9 ± 5.0^‡^ 3-month postoperatively A: 18.6 ± 7.5 B: 36.4 ± 4.7^§^ Last follow-up (≥2-year) A: 21.1 ± 7.9 B: 37.4 ± 4.5^§^	NR	Preop- A: 25.56 ± 9.67 B: 23.66 ± 10.35^‡^ 3-month postoperatively A: 4.92 ± 3.92 B: 18.67 ± 9.64^§^ Last follow-up (≥2-year) A: 6.26 ± 3.39 B: 19.88 ± 10.08^§^	Preop- A: 28.10 ± 8.26 B: 27.02 ± 10.25^‡^ 3-month postoperatively A: 8.03 ± 6.42 B: 18.01 ± 8.68^§^ Last follow-up (≥2-year) A: 8.20 ± 6.49 B: 18.91 ± 8.79^§^	NR
Segmental lordosis	Preop- A: 43.6 ± 9.0° B: 40.7 ± 9.2^°‡^ Last follow-up (≥2-year) A: 48.7 ± 7.3° B: 46.7 ± 8.1^°‡^	NR	NR	NR	Preop- A: 16.66 ± 8.49° B: 13.81 ± 7.51^°‡^ Last follow-up (≥2-year) A: 11.93 ± 7.11° B: 11.36 ± 6.07^°‡^
Good and excellent rate (%)	A: 100 (21/21) B: 100 (13/13)^‡^	NR	A: 85.4 (35/41) B: 86.5 (32/37)^‡^	A: 83.3 (20/24) B: 81.0 (17/21)^‡^	A: 90.0 (27/30) B: 96.2 (25/26)^‡^
Surgery-related complication rate (%)	A: 19.0 (4/21) B: 38.5 (5/13)^‡^	A: 14.81 (24/162) B: 10 (12/120)^‡^	A: 7.3 (3/41) B: 5.4 (2/37)^‡^	A: 4.2 (1/24) B: 0 (0/21)^‡^	A: 10 (3/30) B: 23.1 (6/26)^‡^

A, reduction group; B, *in situ* group; NR, not reported.

^‡^P > 0.05.

^§^P < 0.05.

**Table 3:** Quality evaluation of cohort studies by the NOS scale.

**References**	**Selection**	**Comparability**	**Outcomes**	**Total score (max 9)**
Gong et al. (18)	^**^	^**^	^***^	7
Scheer et al. (19)	^**^	^**^	^**^	6
Fan et al. (20)	^**^	^**^	^***^	7
Fan et al. (21)	^**^	^**^	^***^	7
Tay et al. (22)	^**^	^**^	^***^	7

^*^One score. ^**^Two score.

### Clinical outcome

#### Operation time

**Figure 2:**
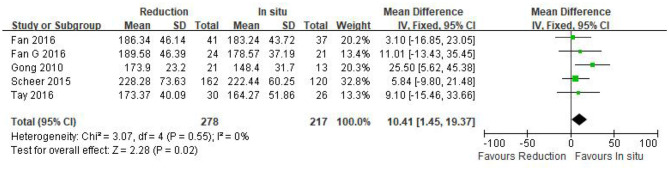
Weighted mean difference of the operation time between the reduction group and the arthrodesis *in situ* group. SD, standard deviation; CI, confidence interval; IV inverse variance.

Five studies (*n* = 495 patients; 278 patients underwent the reduction procedure and 217 were included in the arthrodesis *in situ* group) reported the operation time in the form of mean ± standard deviation. The pooled data showed that the reduction group had a longer operation time than the arthrodesis *in situ* group [*P* = 0.02 < 0.05, WMD 10.41 (1.45, 19.37); Figure 2]. χ^2^ tests showed no statistical evidence of heterogeneity (*P* = 0.55 > 0.05, *I*^2^ = 0%).

#### Intraoperative blood loss

**Figure 3:**
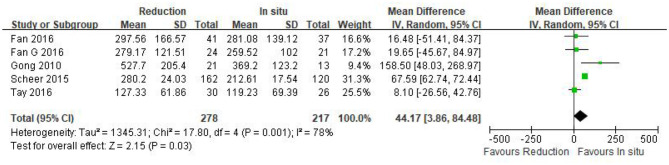
Weighted mean difference of intraoperative blood loss between the reduction group and the arthrodesis *in situ* group. SD, standard deviation; CI, confidence interval; IV, inverse variance.

Intraoperative blood loss was reported in five eligible studies (*n* = 495 patients; 278 in the reduction group and 217 in the arthrodesis *in situ* group). A forest plot of the pooled results indicated more intraoperative blood loss in the reduction group than in the arthrodesis *in situ* group [*P* = 0.03 < 0.05, WMD 44.17 (3.86, 84.48); Figure 3]. However, significant heterogeneity was detected by the chi-squared test (*P* < 0.05, *I*^2^ = 78%).

#### Preoperative slippage and postoperative slippage at short- and long-term follow-up

**Figure 4:**
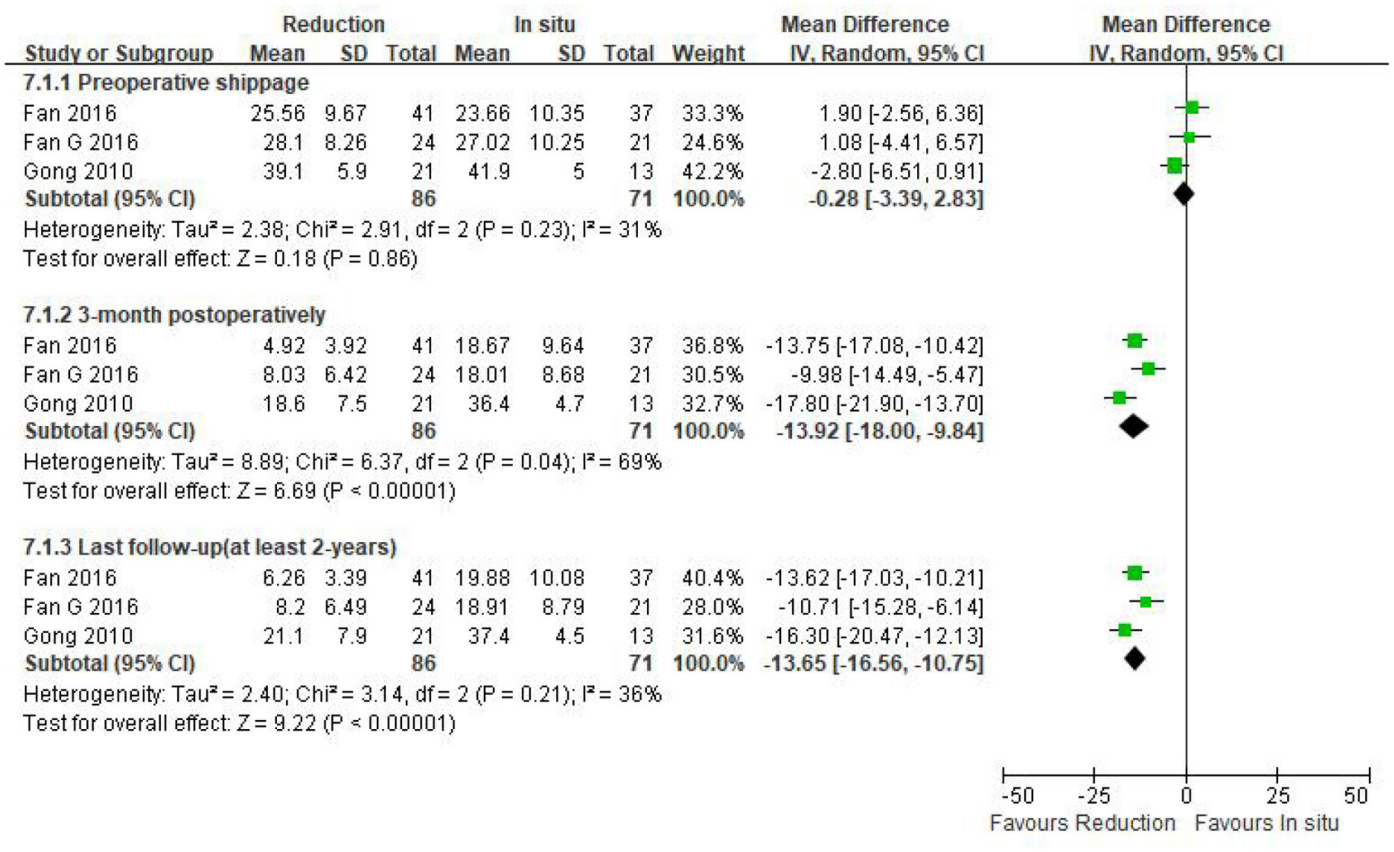
Weighted mean difference of preoperative slippage and postoperative slippage at short- and long-term follow-up between the reduction group and the arthrodesis *in situ* group. SD, standard deviation; CI, confidence interval; IV, inverse variance.

Three eligible studies (*n* = 157 patients; 86 in the reduction group and 71 in the arthrodesis *in situ* group) estimated the slippage degree. In addition, no statistically significant difference was observed in preoperative slippage between both groups [*P* = 0.86 > 0.05, WMD −0.28 (−3.39, 2.83), heterogeneity: Tau^2^ = 2.38, Chi^2^ = 2.91, df = 2, *P* = 0.23, *I*^2^ = 31%; Figure 4].

The same three studies as above (*n* = 157 patients; 86 patients in the reduction group and 71 in the arthrodesis *in situ* group) reported postoperative slippage at short- and long-term follow-up. It is clear that the reduction group was associated with better slippage correction than the arthrodesis *in situ* group during short- and long-term follow-up [*P* < 0.05, WMD −13.92 (−18.00, −9.84), heterogeneity: Tau^2^ = 8.89, Chi^2^ = 6.37, df = 2, *P* = 0.04, *I*^2^ = 69%; *P* < 0.05, WMD −13.65 (−16.56, −10.75), heterogeneity: Tau^2^ = 2.40, Chi^2^ = 3.14, df = 2, *P* = 0.21, *I*^2^ = 36%; respectively; Figure 4].

#### Preoperative VAS-BP and postoperative VAS-BP during short- and long-term follow-up

**Figure 5:**
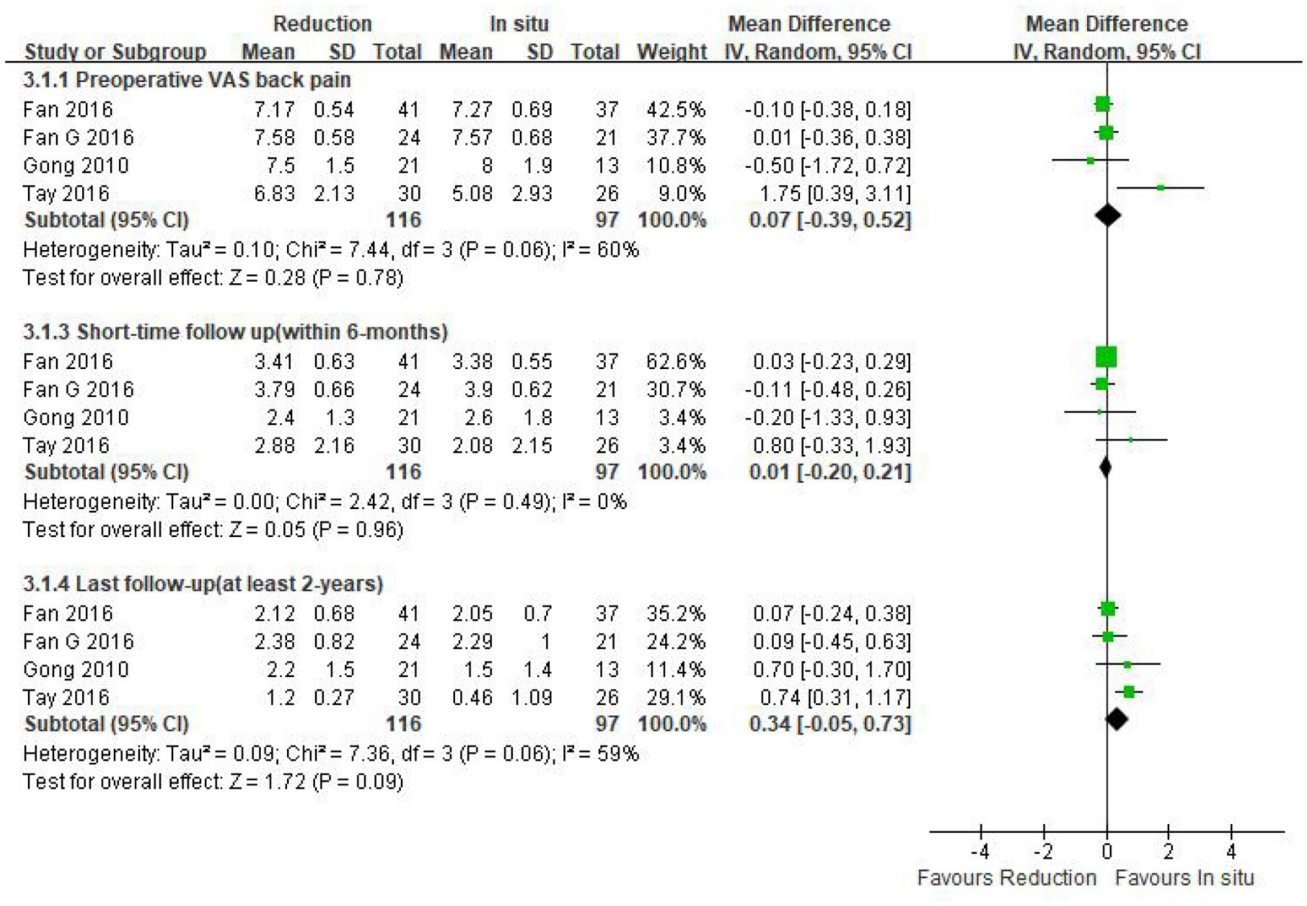
Weighted mean difference of preoperative VAS-BP and postoperative VAS-BP during short- and long-term follow-up between the reduction group and the arthrodesis *in situ* group. SD, standard deviation; CI, confidence interval; IV, inverse variance.

Data regarding preoperative VAS-BP were available in four studies (*n* = 213 patients; 116 patients underwent intraoperative reduction and 97 underwent arthrodesis *in situ*). No statistically significant difference was observed in the preoperative VAS-BP score between both groups [*P* = 0.78 > 0.05, WMD 0.07 (−0.39, 0.52); heterogeneity: Tau^2^ = 0.10, Chi^2^ = 7.44, df = 3, *P* = 0.06, *I*^2^ = 60%; Figure 5].

Four studies mentioned above (*n* = 213 patients; 116 in the reduction group and 97 in the arthrodesis *in situ* group) provided the postoperative VAS-BP score during short- and long-term follow-up. The pooled result indicated that no statistical difference was detected in either short-term or long-term follow-up of VAS-BP between the two groups [*P* = 0.96 > 0.05, WMD 0.01 (−0.20, 0.21), heterogeneity: Tau^2^ = 0.00, Chi^2^ = 2.42, df = 3, *P* = 0.49, *I*^2^ = 0%; *P* = 0.09 > 0.05, WMD 0.34 (−0.05, 0.73), heterogeneity: Tau^2^ = 0.09, Chi^2^ = 7.36, df = 3, *P* = 0.06, *I*^2^ = 59%; respectively; Figure 5].

The above information indicated that the patient-reported outcomes of preoperative and postoperative back pain were similar in both groups.

#### Preoperative ODI and postoperative ODI at short- and long-term follow-up

**Figure 6:**
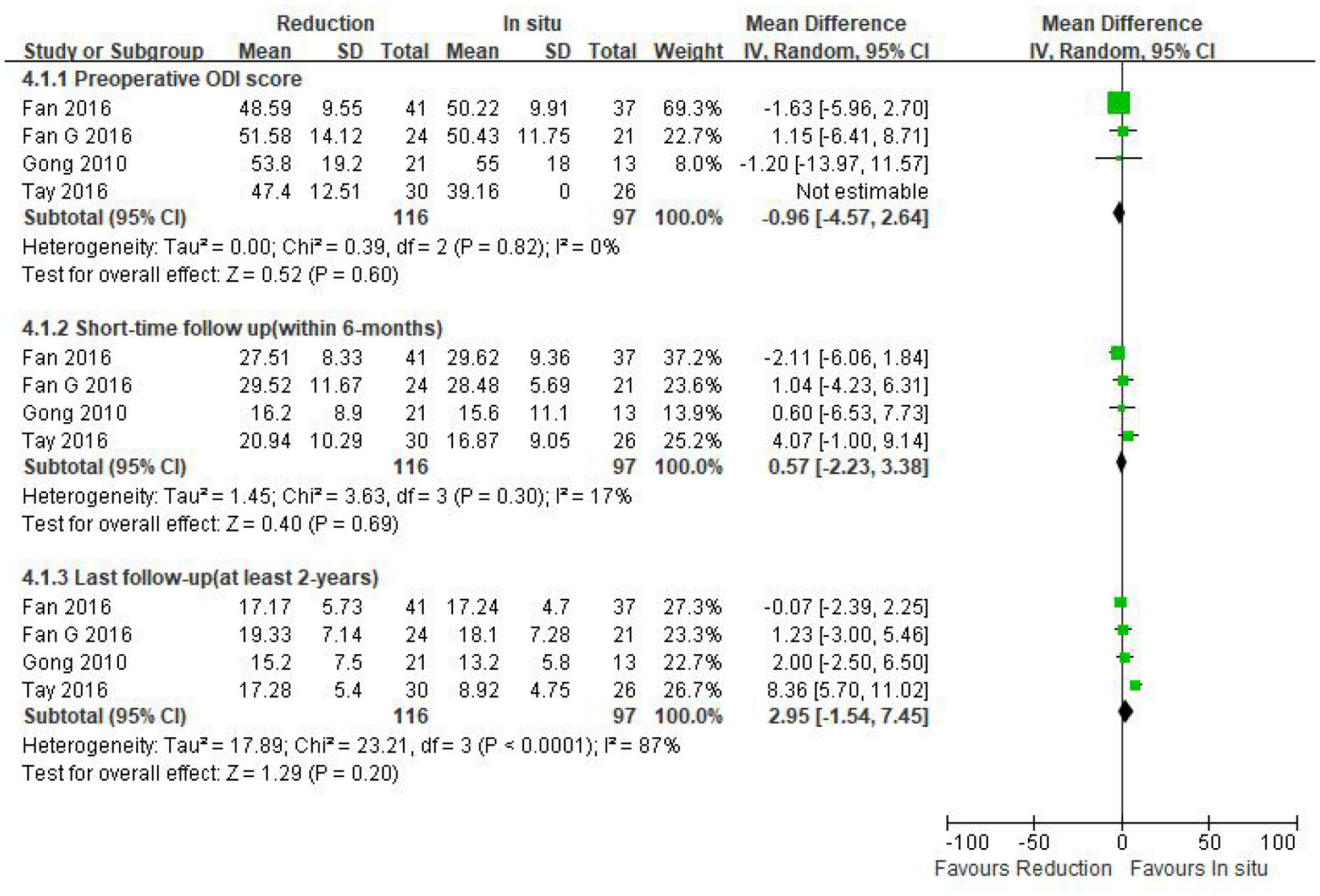
Weighted mean difference of preoperative ODI and postoperative ODI during short- and long-term follow-up between the reduction group and the arthrodesis *in situ* group. SD, standard deviation; CI, confidence interval; IV, inverse variance.

Preoperative ODI was reported in four eligible studies (*n* = 213 patients; 116 patients underwent intraoperative reduction and 97 underwent arthrodesis *in situ*). No significant difference was observed in the preoperative ODI between the two groups [*P* = 0.60 > 0.05, WMD −0.96 (−4.57, 2.64); Figure 6]. Moreover, no statistical heterogeneity was detected by the chi-square test (*P* = 0.82 > 0.05, *I*^2^ = 0%).

The same four studies mentioned above (*n* = 213 patients; 116 in the reduction group and 97 in the arthrodesis *in situ* group) estimated the short- and long-term follow-up of the ODI. In addition, no statistical difference was detected in the postoperative ODI during short- and long-term follow-up between the two groups [*P* = 0.69 > 0.05, WMD 0.57 (−2.23, 3.38), heterogeneity: Tau^2^ = 1.45, Chi^2^ = 3.63, df = 3, *P* = 0.30, *I*^2^ = 17%; *P* = 0.20 > 0.05, WMD 2.95 (−1.54, 7.45), heterogeneity: Tau^2^ = 17.89, Chi^2^ = 23.21, df = 3, *P* < 0.05, *I*^2^ = 87%; respectively; Figure 6].

In summary, the above result indicated that the patient-reported outcomes of both preoperative and postoperative function were similar in both groups.

#### Good and excellent rate

**Figure 7:**
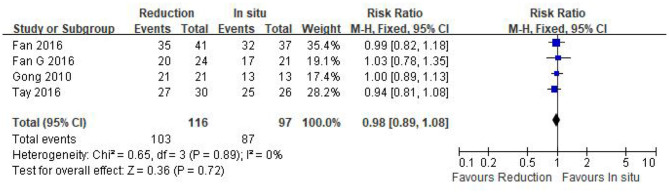
Relative ratio of the good and excellent rate between the reduction group and the arthrodesis *in situ* group. CI, confidence interval; M-H Mantel–Haenszel.

Four eligible studies (*n* = 213 patients; 116 in the reduction group and 97 in the arthrodesis *in situ* group) reported good and excellent rates based on the MacNab criteria (24). The pooled plot shows that no statistical difference was observed in the good and excellent rate between the two groups [*P* = 0.72 > 0.05, RR 0.98 (0.89, 1.08); Figure 7]. χ^2^ tests showed no statistical evidence of heterogeneity (*P* = 0.89 > 0.05, *I*^2^ = 0%).

#### Segmental lordosis

**Figure 8:**
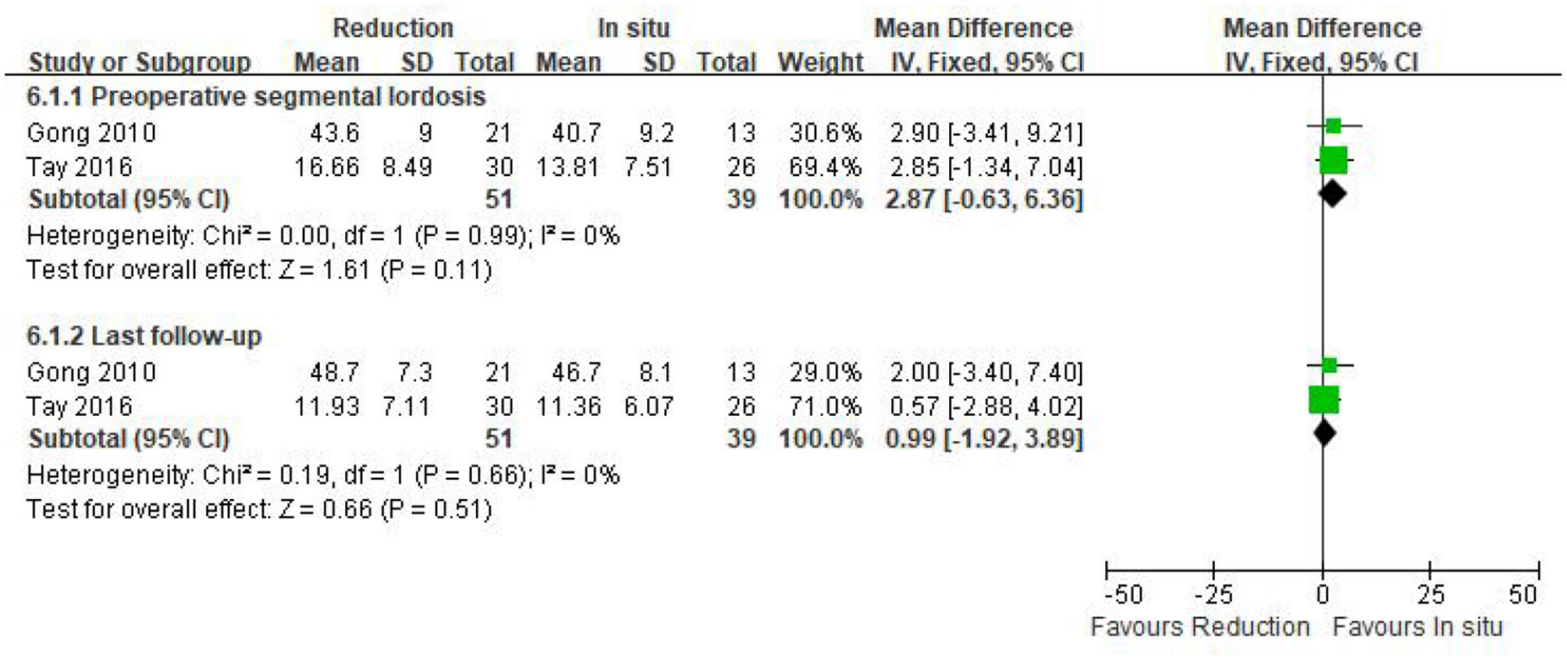
Weighted mean difference of preoperative and last follow-up segmental lordosis between the reduction group and the arthrodesis *in situ* group. SD, standard deviation; CI, confidence interval; IV, inverse variance.

Only two studies (*n* = 90 patients; 51 in the reduction group and 39 in the arthrodesis *in situ* group) estimated segmental lordosis before surgery and at the last follow-up. No statistically significant difference was observed in preoperative and the last follow-up of segmental lordosis between the two groups [*P* = 0.11 > 0.05, WMD 2.87 (−0.63, 6.36); *P* = 0.51 > 0.05, WMD 0.99 (−1.92, 3.89); respectively; Figure 8]. In addition, no statistical heterogeneity was detected by the chi-squared test (*P* = 0.99 > 0.05, *I*^2^ = 0%; *P* = 0.66 > 0.05, *I*^2^ = 0%; respectively).

#### Fusion rate

**Figure 9:**
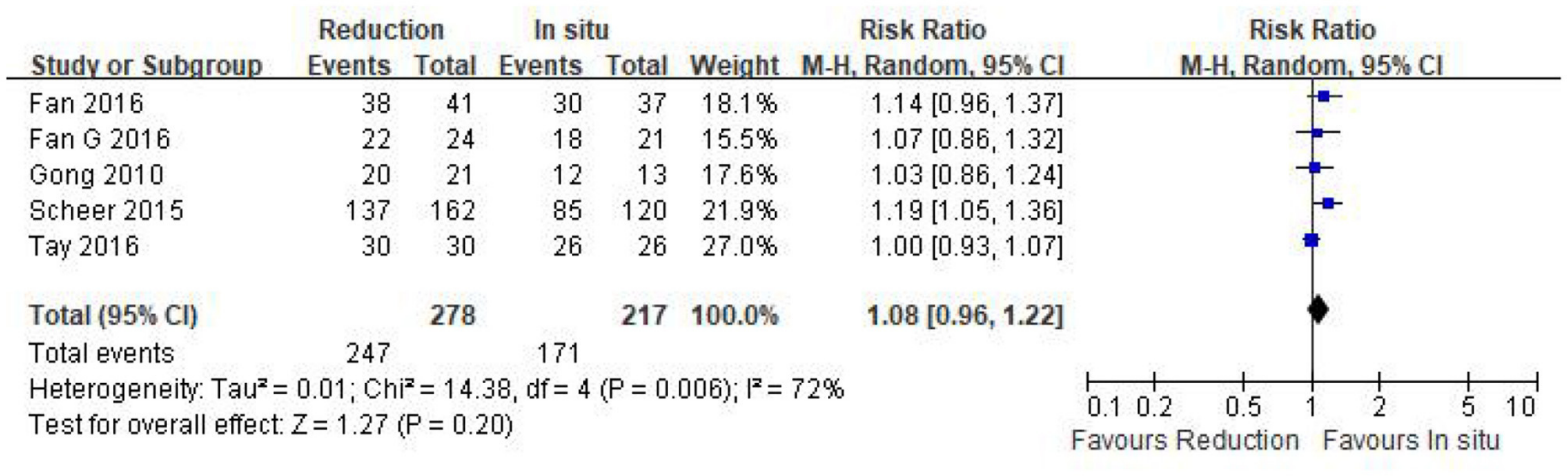
Relative ratio of the fusion rate between the reduction group and the arthrodesis *in situ* group. CI, confidence interval; M-H, Mantel–Haenszel.

Data regarding the fusion rate were available in all of the five studies (*n* = 495 patients; 278 patients underwent intraoperative reduction and 217 underwent arthrodesis *in situ*). No statistical difference was detected in the fusion rate between the two groups [*P* = 0.20 > 0.05, WMD 1.08 (0.96, 1.22); heterogeneity: Tau^2^ = 0.01, Chi^2^ = 14.38, df = 4, *P* < 0.05, *I*^2^ = 72%; Figure 9].

#### Surgery-related complication rate

**Figure 10:**
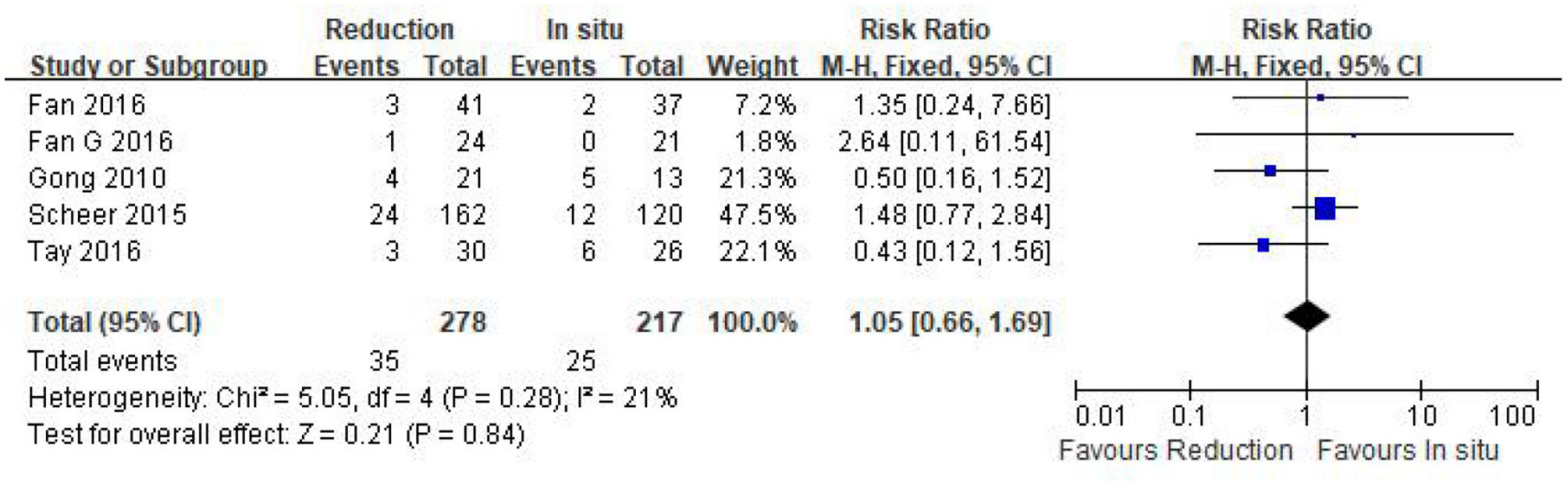
Relative ratio of the surgery-related complication rate between the reduction group and the arthrodesis *in situ* group. CI, confidence interval; M-H, Mantel–Haenszel.

The surgery-related complication rate was assessed in five eligible studies (*n* = 495 patients; 278 in the reduction group and 217 in the arthrodesis *in situ* group). The pooled result indicated that no statistical difference was observed in the surgery-related complication rate between the two groups [*P* = 0.84 > 0.05, RR 1.05 (0.66, 1.69); heterogeneity: Chi^2^ = 5.05, df = 4, *P* = 0.28, *I*^2^ = 21%; Figure 10].

## Discussion

The initial surgical strategy for lumbar spondylolisthesis was laminectomy, followed by lumbar interbody fusion. The implementation of fusion surgery can effectively improve the clinical prognosis in patients with symptomatic lumbar spondylolisthesis. Compared with PLIF, the TLIF technique reduces the retraction of the thecal sac and nerve roots and preserves the structural integrity of the posterior column, which reduces the risk of various complications, including neurological tissue damage. In addition, interbody fusion can maintain the height of the intervertebral space and restore the height of the intervertebral foramen, thereby improving or relieving the narrowing of the intervertebral foramen, and the normal anatomical curvature of the corresponding segment can be reconstructed to restore or maintain the overall physiological curvature of the spine. However, whether intentional reduction with TLIF is necessary for the surgical treatment of low-grade lumbar spondylolisthesis remains controversial. Only one meta-analysis study (25) has been performed on this subject, which concluded that no statistical difference was observed in terms of operation time, estimated blood loss, patient-reported outcomes, and fusion rate and complication rate. However, in the aforementioned study, patients in one included study underwent posterolateral fusion (PLF) (26), patients in three studies underwent PLIF (27–29), and patients in the remaining three studies underwent MIS/open-TLIF (18, 21, 22). The uncontrolled confounding factors for surgical operations might exert an effect on their results. To our knowledge, several surgical techniques have been performed to treat symptomatic spondylolisthesis, which could not be treated by conservative treatment strategies. Moreover, the advent of minimally invasive technologies for spine surgery led to the logical progression of open-TLIF to MIS-TLIF (30). The TLIF technique has been proven to be an effective management strategy for spondylolisthesis as it preserves the structural integrity of the posterior column and reduces the retraction of thecal sac and nerve roots (12, 13). In addition, our study aims to compare the clinical efficacy and safety of reduction vs. arthrodesis *in situ* with only the MIS/open-TLIF technique for low-grade spondylolisthesis, as, currently, there is still a lack of powerful clinical evidence. The pooled results in our study revealed that both techniques had similar patient-reported outcomes (VAS, ODI, and good and excellent rate) during short- and long-term follow-up, and no significant difference was observed in the fusion and complication rate, as well as the segmental lordosis. Although the reduction group did achieve better slippage correction, it was associated with increased operation time and intraoperative blood loss compared with the arthrodesis *in situ* group.

Intentional reduction of the slipped vertebrae may be a useful procedure, and the idea remains attractive in theory. It restores the spinal column into a more anatomic alignment. Fan reported that significant advantages in slippage decrease were observed in the reduction group (20). And, as shown in our study, the reduction group did achieve better slippage correction. In addition, some studies considered that intraoperative reduction might relieve early muscular fatigue and hypolordosis-induced back pain and prevent disc degeneration of the adjacent segment (31, 32). However, no evidence-based conclusion was reached to support the above viewpoint (29, 33). The pooled plot in our study indicated that the reduction group had increased operation time and blood loss, which might seem reasonable, especially in MIS-TLIF technology.

Patient-reported outcomes such as VAS-BP and ODI are of paramount importance when estimating the clinical effect of a certain technique (34). We predefined a follow-up that ranged from 3 to 6 months after surgery as a short-term follow-up and a follow-up of at least 2 years as a long-term follow-up. In our study, both techniques had similar VAS-BP and ODI during short- and long-term follow-up. The primary goal of intervention for spinal diseases is to relieve the pain and restore function (35, 36), and the excessive pursuit of anatomic restoration in MIS/open-TLIF procedures may not help in improving pain and function in patients (20). In addition, for the complication and fusion rate, Bai et al.'s (25) paper of meta-analysis demonstrated no statistical difference, which was again confirmed by our findings. In a word, intraoperative reduction in MIS/open-TLIF procedures, while safe, did not result in better clinical outcomes in patients with low-grade lumbar spondylolisthesis.

Moreover, bone quality is an important factor in the success of the reduction of slippage in the vertebrae. It is not easy to obtain the reduction, especially in patients with osteoporosis. Surgeons may fail in intraoperative reduction due to screw loosening in low-bone mass patients. However, the index of bone density was not mentioned in all five included studies in our meta-analysis. For intraoperative screw loosening after reduction, bone cement could be used to reinforce the pedicle screws.

We recognize the limitations of our study. First, all studies included in this meta-analysis were retrospective cohort studies and were inherently prone to methodology defects. Although all studies were rated with a total score of more than 5 according to the NOS (23), which represented relatively moderate to high quality, the validity of the data available might be weakened due to selection bias and other biases. In addition, the quality of evidence based on GRADE (37) was very low (Table 4), and the strength of the recommendations was relatively weak. Second, a relatively small number of participants and various definitions of complications among the studies might exert an effect on the results because of the limited statistical power and homogeneity. Third, the decision for intraoperative reduction was made primarily based on surgeon preference, and diverse MIS/open-TLIF technical specifications and postoperative management applied by diverse surgeons in different treatment centers might also have influenced the results. Moreover, data about health-related quality of life (HRQOL) were not available, which would be necessary to know if patients undergoing intraoperative reduction had an improvement in the HRQOL score over the patients who underwent arthrodesis *in situ*.

**Table 4:** Quality evaluation according to GRADE.

**References**	**Published year**	**Risk of bias**	**Indirectness**	**Imprecision**	**Publication bias**	**Large effect**	**Plausible residual confounding**	**Total**	**Quality of evidence**
Gong et al. (18)	2015	−1	0	N/A	−1	0	0	−2	Very low
Scheer et al. (19)	2013	−1	0	N/A	−1	0	0	−2	Very low
Fan et al. (20)	2012	−1	0	N/A	−1	0	0	−1	Very low
Fan et al. (21)	2007	−1	0	N/A	−1	0	0	−2	Very low
Tay et al. (22)	2008	−1	0	N/A	−1	0	0	−2	Very low

## Conclusion

Based on the available evidence, intraoperative reduction does not result in better clinical outcomes in low-grade spondylolisthesis after MIS/open-TLIF, and the arthrodesis *in situ* technique could be an alternative. In view of the limitations of this study, a multicenter, large sample, well-designed randomized controlled study is essential to draw a more convincing conclusion.

## Data availability statement

The original contributions presented in the study are included in the article/supplementary material, further inquiries can be directed to the corresponding author.

## Author contributions

RQ: Validation, Writing – original draft, Writing – review & editing. MZ: Data curation, Investigation, Methodology, Software, Writing – review & editing. PZ: Software, Supervision, Validation, Writing – review & editing. AG: Software, Supervision, Validation, Writing – review & editing.
